# Regulation of neuraminidase expression in *Streptococcus pneumoniae*

**DOI:** 10.1186/1471-2180-12-200

**Published:** 2012-09-11

**Authors:** Luciana Gualdi, Jasvinder Kaur Hayre, Alice Gerlini, Alessandro Bidossi, Leonarda Colomba, Claudia Trappetti, Gianni Pozzi, Jean-Denis Docquier, Peter Andrew, Susanna Ricci

**Affiliations:** 1 Dipartimento di Biotecnologie, Università di Siena, Siena, Italy; 2 Department of Infection Immunity and Inflammation, University of Leicester, Leicester, United Kingdom; 3 UOC Batteriologia, Azienda Universitaria Ospedaliera Senese, Siena, Italy; 4 LAMMB, Policlinico Le Scotte lotto 5 piano 1, Siena, 53100, Italy; 5 Research Centre for Infectious Diseases, School of Molecular and Biomedical Science, University of Adelaide, South Australia, 5005, Australia

**Keywords:** Sialic acid, Metabolic regulation, Carbon catabolite repression

## Abstract

**Background:**

Sialic acid (N-acetylneuraminic acid; NeuNAc) is one of the most important carbohydrates for *Streptococcus pneumoniae* due of its role as a carbon and energy source, receptor for adhesion and invasion and molecular signal for promotion of biofilm formation, nasopharyngeal carriage and invasion of the lung.

**Results:**

In this work, NeuNAc and its metabolic derivative N-acetyl mannosamine (ManNAc) were used to analyze regulatory mechanisms of the neuraminidase locus expression. Genomic and metabolic comparison to *Streptococcus mitis, Streptococcus oralis, Streptococcus gordonii* and *Streptococcus sanguinis* elucidates the metabolic association of the two amino sugars to different parts of the locus coding for the two main pneumococcal neuraminidases and confirms the substrate specificity of the respective ABC transporters. Quantitative gene expression analysis shows repression of the locus by glucose and induction of all predicted transcriptional units by ManNAc and NeuNAc, each inducing with higher efficiency the operon encoding for the transporter with higher specificity for the respective amino sugar. Cytofluorimetric analysis demonstrated enhanced surface exposure of NanA on pneumococci grown in NeuNAc and ManNAc and an activity assay allowed to quantify approximately twelve times as much neuraminidase activity on induced cells as opposed to glucose grown cells.

**Conclusions:**

The present data increase the understanding of metabolic regulation of the *nanAB* locus and indicate that experiments aimed at the elucidation of the relevance of neuraminidases in pneumococcal virulence should possibly not be carried out on bacteria grown in glucose containing media.

## Background

*Streptococcus pneumoniae* is a common inhabitant of the upper respiratory tract and it is also a major human pathogen. The self-limited carriage episodes represent the most common interaction between pneumococci and the host. However, in some cases, such asymptomatic interaction can progress to invasive disease [1]. Of the many factors influencing the interaction of the bacterium with the host, numerous extracellular glycosyl-hydrolases and carbohydrate transporters have been found to play significant roles [2]. The sialidases or neuraminidases, which are able to cleave terminal sialic acid (neuraminic acid, NeuNAc) residues present in O-linked and N-linked glycans, have since long received special attention as virulence determinants [3,4]. Direct interaction of the microbial sialidases with host glycoproteins resulting in exposure of additional attachment sites on host cells was the mechanisms most frequently found to be involved in virulence [5-7]. Recently such interaction was found to be directly involved in invasion [8,9]. Despite the impact of sialidases in pneumococcal pathogenesis, metabolic implications have received less attention, including the utilisation of sialic acid as a carbon source on the glucose-free mucosal surfaces [10-16]. Sialic acid has recently been described by us and others to act as a molecular signal for pneumococci, resulting in increased carriage and translocation of bacteria to the lung [10,14,17].

Given the prominent role of sialidases in host-pathogen interaction, it is not surprising that pneumococci harbour three sialidases, two of which, NanA and NanB, are common to all pneumococci and the third, NanC, is present in only 51% of strains [18]. Structural and functional analysis of the three enzymes indicated possible different roles. NanA is a first-line virulence factor for sialic acid removal, the trans-sialidase NanB is involved in the metabolic use of sialic acid, and NanC has a regulatory role, being able to produce and remove an intermediate metabolic compound which also acts as sialidase inhibitor [19,20]. The conserved *nanAB* locus that comprises the genes between SPG1583 and SPG1601 in strain G54 (SP1674-94 in TIGR4) was identified as the cluster responsible for uptake and metabolism of sialic acid [16,21-23]. In addition to the extracellular sialidases NanA and NanB, the regulon encodes two ABC transporters, one of which responsible for sialic acid and N-acetyl mannosamine uptake SPG1589-91 (*satABC)* and the other (SP1596-8) for uptake of N-acetyl mannosamine alone [14,23]. In addition to the ABC transporters the locus encodes a PTS uptake system for glucosamine, and the remaining genes encode for enzymes involved in sialic acid metabolism [23]. *In vitro* this operon was found to be the main cluster showing differential expression in pneumococcal opacity variants and was predicted to be composed of four predicted transcriptional units [21]. During infection, the *nanAB* operon was found to be upregulated in pneumonia and meningitis compared to growth in blood [24,25]. Much less information is available on the *nanC* operon, except for the analysis of the enzymatic function of the sialidase NanC [20] and its recent implication as an alternative system for the uptake of sialic acid [23].

The present work aims at performing a functional analysis of the operon in order to gain further insight into the metabolic regulation of this locus.

## Results

### The NanAB locus conservation in oral streptococci

**Figure 1:**
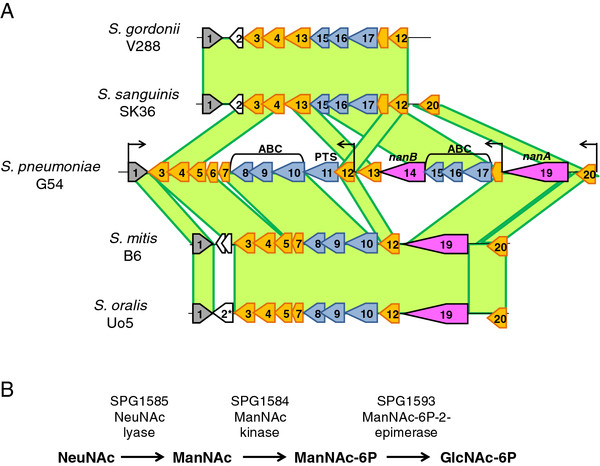
**Structure of the neuraminidase locus in different streptococci. A**. The schematic maps of the *nanAB* operon of *S. pneumoniae* G54 and the orthologous locus in its close relatives, including *S. gordonii* V288 (NC_009785.1), *S. sanguinis* SK36 (NC_009009.1) [46], *S. mitis* B6 (NC_013853.1) [47] and *S. oralis* Uo5 (NC_015291.1) [48] are shown. In *S. pneumoniae* the complete locus includes 18 ORFs, some of them conserved in the other species [23]. The two neuraminidases (NanA and NanB) are in pink, while the three different transporters (two ABC transporters and one PTS) are in blue. The phosphosugar binding transcriptional regulator is shown in grey and the metabolic enzymes involved in sialic acid metabolism are in orange. The homologous regions in green refer to DNA identity above 50% and represent orthology of genes. The black arrows placed upstream of *SPG1601*, SPG1599, SPG1593, and SPG1583 represent the promoters of the regulon [21]. The gene numeration is detailed in Table 1. **B**. Schematic representation of the first steps in sialic acid catabolism. The first step involves the N-acetylneuraminate lyase SPG1585 which removes a pyruvate group from sialic acid, yielding N-acetylmannosamine (ManNAc). Subsequently, an N-acetylmannosamine kinase (SPG1584) adds a phosphate group to ManNAc, resulting in the formation of N-acetylmannosamine-6-phosphate (ManNAc-6P). SPG1593 encodes an N-acetylmannosamine-6-phosphate 2-epimerase, which transforms ManNAc-6P into N-acetylglucosamine-6-phosphate (GlcNAc-6P) [15,16].

**Figure 2:**
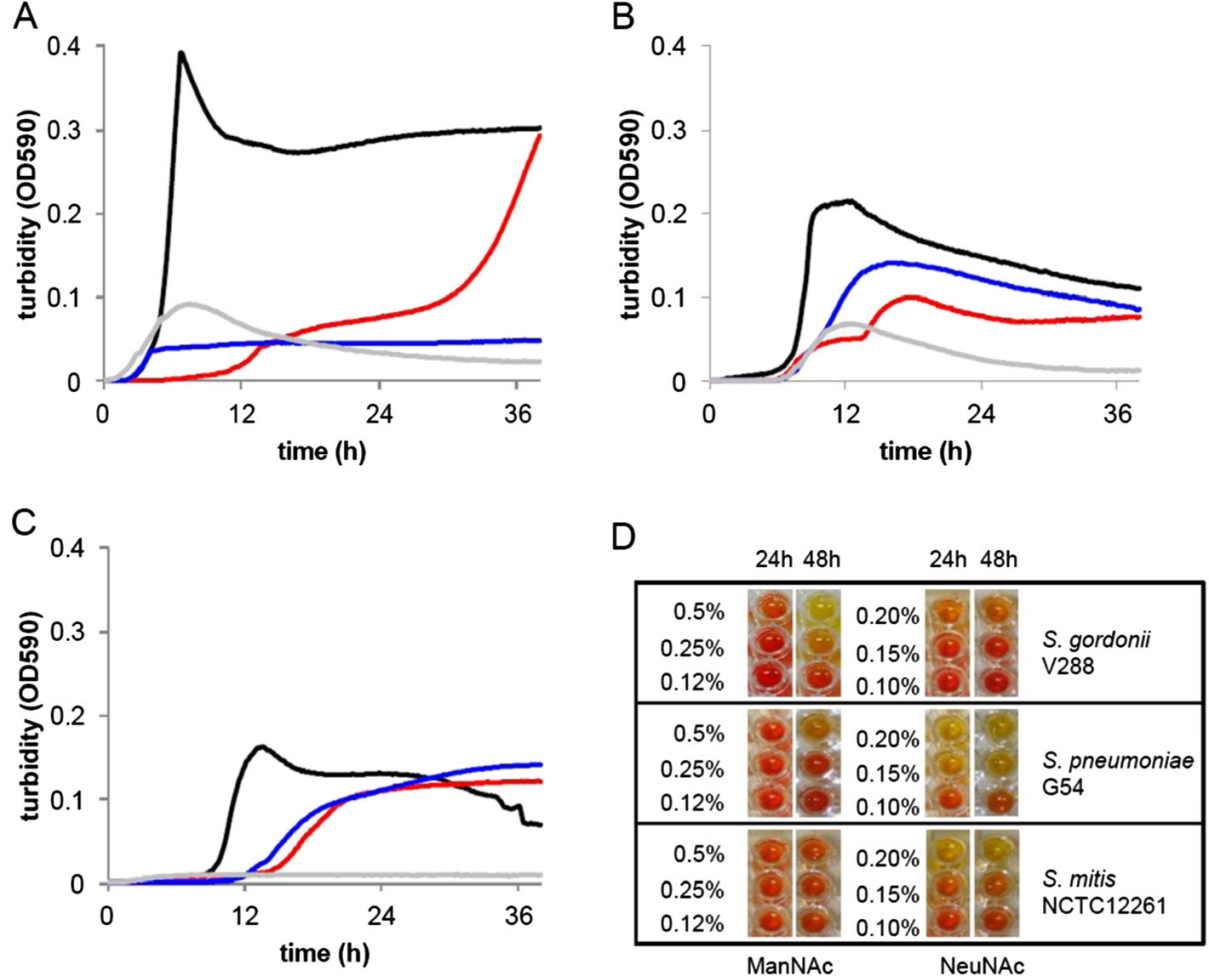
**Metabolic utilisation 0f ManNAc and NeuNAc by *S. gordonii, S. mitis *and *S. pneumoniae *. ***S. gordonii* V288 (**A**), *S. pneumoniae* G54 (**B**), and *S. mitis* NCTC12661 (**C**) were grown in CAT medium (200 U catalase) supplemented with 2 g/L glucose (black line) 2 g/L ManNAc (red line) and 1.5 g/L NeuNAc (blue line). CAT medium alone as a source of carbon is in grey line. All strains were grown for 38 hours at 37°C in 200 μl of medium in a 96 well microplate with reading intervals of 10 min. For the fermentation assay (panel **D**) bacteria were incubated for 24 and 48 h with serial dilutions of either ManNAc (left columns) or NeuNAc (right columns) as sole carbon sources in microtiter plates containing phenol red as a pH indicator. Sugar fermentation is evidenced by a yellow colour change due to acidification of the culture medium. Carbohydrate concentrations (% w/v) are shown on the right.

As a first approach to elucidate the metabolic relevance and regulation of the different predicted transcriptional units of the *nanAB* regulon, we performed a genomic comparison amongst related streptococcal species, including pneumococcal strain G54, *S. mitis* B6, *S. oralis* Uo5, *S. sanguis* SK36 and *S. gordonii* V288 (Figure 1A and Table 1). With respect to *S. pneumoniae* G54, *S. mitis* B6 and *S. oralis* Uo5, these showed an identical organization for part of the locus including the neuraminidase A (*nanA*), the orthologs of the *satABC* transporter SPG1589-91 and the genomic regions encoding the transcriptional regulator and orthologues of the enzymes involved in the first steps of sialic acid metabolism, i.e. N-acetylneuraminate lyase and N-acetylmannosamine kinase (Figure 1). In contrast to pneumococci these two species, *S. mitis* and *S. oralis*, did not possess the sialidase NanB, the second ABC transporter SPG1596-8, and the PTS system. In contrast to *S. mitis* and *S. oralis*, *S. gordonii* V288 and *S. sanguinis* SK36 did not possess any neuraminidases. Interestingly both *S. gordonii* and *S. sanguis* still possess orthologs of the N-acetylneuraminate lyase, N-acetylmannosamine kinase and N-acetylmannosamine-6-phosphate 2-epimerase predicted to be necessary for metabolism of sialic acid (Figure 1A,B; Table 1). In addition, *S. gordonii* and *S. sanguis* possessed the transcriptional regulator and the orthologs of the pneumococcal SPG1596-8 ABC transporter. In contrast to *S. pneumoniae*, *S. gordonii* and *S. sanguis* possess neither the PTS system nor the SPG1589-91 *satABC* transporter. To check the amino sugar metabolism of these three different species of streptococci growth curves and fermentation assay on NeuNAc and ManNAc were performed. The growth curves show that *S. gordonii* grows only in presence of ManNAc, while *S. mitis* and *S. pneumoniae* are capable of growth on both amino sugars (Figure 2A,C). Similarly in the fermentation assay only *S. gordonii* acidified efficiently the medium in presence of ManNAc, while both *S. pneumoniae* and S*. mitis* metabolised efficiently only NeuNAc, with some acidification of the medium with ManNAc by the pneumococcus (Figure 2D).

**Table 1:** **List of gene annotation in the *nanAB *locus**

**Annotation**	**Figure **1A*	***S. pneumoniae TIGR4***	***S. pneumoniae *G54**	***S. mitis B6***	***S. oralis Uo5***	***S. gordonii V288***	***S. sanguinis SK36***
Regulator	1	SP1674	SPG1583	smi0612	SOR0560	SGO0127	SSA0081
Hypothetical protein	2	-	-	smi0610	SOR0559	SGO0126	SSA0080
N-acetylmannosamine kinase	3	SP1675	SPG1584	smi0609	SOR0558	SGO0125	SSA0079
N-acetylneuraminate lyase	4	SP1676	SPG1585	smi0608	SOR0557	SGO0124	SSA0078
hypothetical protein	5	SP1677	SPG1586	smi0607	SOR0556	-	-
hypothetical protein	6	SP1679	-	-	-	-	-
hypothetical protein	7	SP1680	SPG1588	smi0606	SOR0555	-	-
*satA* ABC transporter permease	8	SP1681	SPG1589	smi0605	SOR0553	-	-
*satB* ABC transporter permease	9	SP1682	SPG1590	smi0604	SOR0552	-	-
*satC* ABC transporter substrate-binding protein	10	SP1683	SPG1591	smi0603	SOR0550	-	-
PTS system, IIBC components	11	SP1684	SPG1592	-	-	-	-
NanE, ManAc-6P 2-epimerase	12	SP1685	SPG1593	smi0602	SOR0549	SGO0118	SSA0071
oxidoreductase	13	SP1686	SPG1594	-	-	SGO0123	SSA0077
NanB neuraminidase	14	SP1687	SPG1595	-	-	-	-
ABC transporter permease	15	SP1688	SPG1596	-	-	SGO0122	SSA0076
ABC transporter permease	16	SP1689	SPG1597	-	-	SGO0121	SSA0075
ABC transporter substrate-binding protein	17	SP1690	SPG1598	-	-	SGO0120	SSA0074
hypothetical protein	18	SP1691	SPG1599	-	-	SGO0119	SSA0073
NanA neuraminidase	19	SP1693	SPG1600	smi0601	SOR0548	-	-
Acetyl xylan esterase	20	SP1694	SPG1601	smi0600	SOR0547	-	SSA0070

* numbers as in Figure 1A.

### Neuraminidase locus induction in *S. pneumoniae*

**Figure 3:**
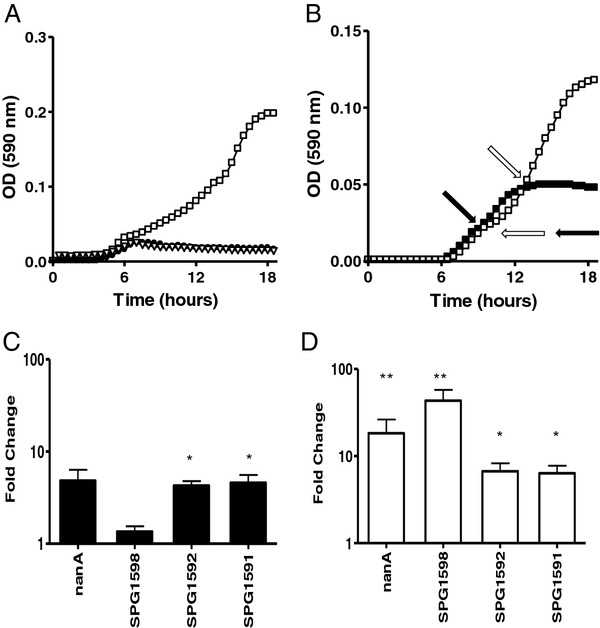
**Growth and induction of gene expression by ManNAc.** (**A**) Growth of *S. pneumoniae* strains on CAT medium supplemented with 10 g/L of ManNAc: FP65 (open squares), *nanAB*-deficient mutant (open triangles), and SPG1583-regulator deletion mutant (closed circles). (**B**) Growth of FP65 on CAT medium without added sugar (closed squares) and supplemented with ManNAc 10 g/L (open squares). The white and black arrows indicate samples taken for quantitative Real Time-PCR. Gene expression analysis of the genes coding for NanA the ABC transporter SPG1598, the PTS transporter SPG1592, and the ABC transporter SPG1591 is shown in panel **C** and **D**. Panel **C** refers to fold changes in transcriptional levels at OD 0.02 in medium with or without ManNAc (for sampling see closed arrows in panel 3**B**). Panel **D** refers to analysis of sequential samples (OD_590_ = 0.02 and OD_590_ = 0.05) of bacteria grown in ManNAc (for sampling see open arrows in panel 3**B**). The fold changes are reported as mean from independent triplicate or quadruplicate experiments. Two-tailed Student t test was used for analyse statistical significance (*, p < 0.05; **, p < 0.01). Generation time on unsuplemented CAT medium is 40 min and on ManNAc 140 min.

The putative regulator of the *nanAB* locus SPG1583 contains a classical N-terminal helix-turn-helix motif and a SIS domain, found in many phosphosugar binding proteins including transcriptional regulators binding to the phosphorylated end-products of the pathways [26]. Given the probable catabolic pathway of sialic acid (Figure 1B), ManNAc-6-phosphate appears to be the most probably compound having a regulatory role on the expression of pneumococcal neuraminidase operon and thus possibly in sialic acid metabolism [23]. Therefore we analysed the growth curves and the expression levels of some key genes associated with the transporter systems in the neuraminidase locus. First we compared the growth in the presence of ManNAc as a carbon source of a un-encapsulated G54 derivative FP65 and two isogenic mutants devoid of the whole *nanAB* locus and of the transcriptional regulator SPG1583 respectively (Figure 3A). The growth curves showed absence of growth in the presence of ManNAc for both mutants, indicating that the *nanAB* locus is essential for efficient growth of ManNAc and that the phosphosugar binding regulator SPG1583 gene appears to acts as a transcriptional activator. Then we focused our attention on growth of the wild type strain in the presence or absence of ManNAc, preferred by us for the indication assays over NeuNAc, as this amino sugar does not acidify the medium. In these experiments bacteria initially grew on residual yeast-extract derived dextran of non-supplemented CAT medium (40 min) and continued to grow thereafter with a lower generation time of 140 min on ManNAc only (Figure 3B). For gene expression profiling bacteria were sampled in early exponential growth (OD_590_ = 0.02), when growth was still due to the residual yeast extract-derived sugar (Figure 3B, black arrows). For bacteria grown on yeast extract derived sugar in presence of ManNAc, gene expression data showed a significant induction of the *satABC* SPG1589-91 and SPG1592 PTS transporters, and a non-significant induction of *nanA* (Figure 3C). We performed a second experiment that compared the influence of ManNAc at OD_590_ = 0.02 and 0.05 on gene expression (Figure 3B, open arrows). This assay compared bacteria grown on yeast-derived sugar and ManNAc, where the first time point represents growth on yeast-extract derived sugar (in presence of ManNAc) and the second growth on ManNAc after termination of the other carbohydrates. Growth on ManNAc caused a significant increase of transcriptional levels of all genes analysed (Figure 3D). The values of mean fold changes were 17.61 (p < 0.01) for *nanA,* 52.18 (p < 0.01) for SPG1598, 6.33 (p < 0.05) for SPG1592 and 6.65 (p < 0.05) for *satC* SPG1591.

**Figure 4:**
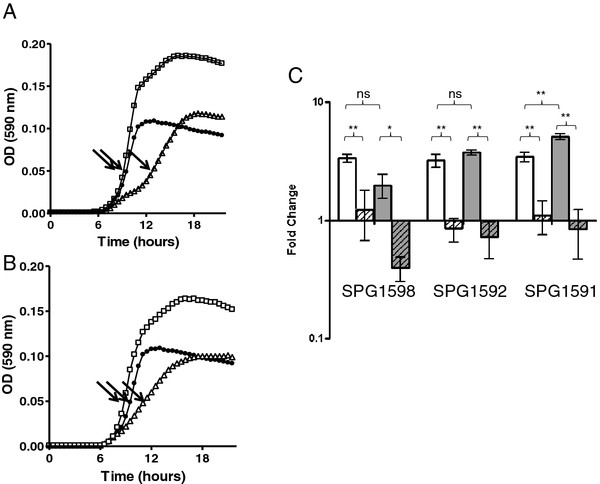
**Repression of *nanAB *locus by glucose.** (**A**) Growth curves of FP65 in medium supplemented with glucose (closed circles), ManNAc (open triangles), and glucose plus ManNAc (open squares). (**B**) Growth curves of PF65 in medium supplemented with glucose (open circles), NeuNAc (open triangles), and glucose plus NeuNAc (open squares). The arrows indicated sampling. (**C**) Gene expression of SPG1598, SPG1592, and SPG1591 in medium supplemented with amino sugars are compared to growth in glucose. Variation of gene expression is shown for genes of bacteria grown in ManNAc (open bars), glucose plus ManNAc (open striped bars), NeuNAc (grey bars), and glucose plus NeuNAc (grey striped bars). Results are represented as fold changes ± SD of gene expression from 3 to 4 independent experiments. Statistical analysis was carried out using Tukey’s Multiple Comparison Test (ns non significant; *, p < 0.05; **, p < 0.01). Generation time on glucose containing media is 38–45 min, 90 min on NeuNAc and 140 min on ManNAc.

To evaluate the role of glucose and of the two amino sugars ManNAc and NeuNAc in the regulation of the *nanAB* regulon, we quantify gene expression during growth in the presence of these sugars. Bacteria were grown in the presence of ManNAc (Figure 4A, open triangles) or NeuNAc (Figure 4B, open triangles) and their gene expression was compared to that of bacteria grown with 1 g/L glucose alone (Figure 4A,B, closed circles). All genes of the *nanAB* regulon showed a significant increase in transcription in presence of any of the amino sugars. The values of mean fold changes were: *nanA,* 2.69 (p ≤ 0.05) in ManNAc and 5.14 (p ≤ 0.05) in NeuNAc; SPG1598, 3.35 (p ≤ 0.05) in ManNAc and 1.99 in NeuNAc; SPG1592, 3.21 (p ≤ 0.05) in ManNAc and 3.74 (p ≤ 0.05) in NeuNAc; SPG1591, 3.45 (p ≤ 0.05) in ManNAc and 5.13 (p ≤ 0.01) in NeuNAc. Interestingly the transporter SPG1596-8 linked to the growth and fermentation of ManNAc was more induced by this sugar, while NeuNAc had a significantly greater effect on the *satABC* SPG1589-91 transporter, again in accordance with phenotypic data.

### Repression of the *nanAB* locus in the presence of glucose

According to the presence of three *cre* sites within the pneumococcal neuraminidase locus, we observed a biphasic growth curve when bacteria grew on glucose plus ManNAc or NeuNAc (Figure 4A,B, open squares). To demonstrate that this phenotype was due to carbon catabolite repression, we investigated the transcriptional behaviour of the neuraminidase locus in the presence or absence of glucose in the medium. Growth conditions used were as follows: ManNAc with and without glucose (Figure 4A, open triangles and open squares), NeuNAc with and without glucose (Figure 4B, open triangles and open diamonds) and glucose as the sole carbon source as a reference condition (Figure 4A and 4B, closed circles). Growth curve data show that addition of glucose to both ManNAc and NeuNAc resulted in an initial growth on glucose as a preferred carbon source followed by a second slower growth phase, in which the amino sugars were metabolised. To assess glucose repression during growth on glucose gene expression analysis was carried out by sampling the bacteria at an OD_590_ of 0.05 (Figure 4A,B, arrows). As shown in Figure 4C, the over-expression of all genes of the *nanAB* locus occurred during growth on ManNAc or NeuNAc as the sole carbon sources (Figure 4C, open and grey bars), while it was completely repressed in the presence of glucose (Figure 4C, striped bars).

### Regulation of neuraminidase A production and activity by ManNAc

**Figure 5:**
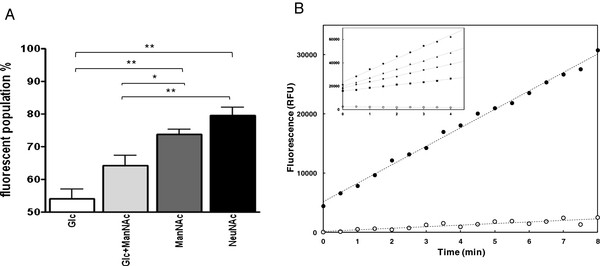
**Neuraminidase protein production and activity on whole cells.** A cytofluorimetric assay with an anti-NanA serum was performed on pneumococci grown on different carbohydrates (panel **A**). The presence of NanA at the bacterial surface was tested in samples cultivated in glucose (open bar), glucose + ManNAc, ManNAc alone (grey bar), and NeuNAc alone (black bar) (all carbohydrates were at 1 g/L). Data are represented as mean values ± SD of percent bacterial population positive for NanA production and derived from quadruplicates experiments performed independently. Asterisks (*, p < 0.05; **, p < 0.001) indicated statistical significance. Panel **B** shows the hydrolysis of 2’-(4-Methylumbelliferyl)-α-D-N-acetylneuraminic acid (4MU-Neu5Ac) in the presence of 40 μl *S. pneumoniae* FP65 cell samples grown in CAT medium with either glucose (white circles) or N-acetylmannosamine (black circles). The neuraminidase activity was computed as the variation of fluorescence vs time using a linear regression of the data (dashed lines). Inlet. Hydrolysis of 4MU-Neu5Ac by purified NanA neuraminidase, showing the proportionality between enzyme concentration and rate of fluorescence variation. Enzyme concentrations were 10 nM (black circles), 20 nM (triangles), 30 nM (diamonds) and 40 nM (squares). The empty circles show the variation of fluorescence *vs* time for the substrate alone.

To assess the production of NanA on the bacterial surface after induction of the *nanAB* locus by ManNAc or NeuNAc, we performed a cytofluorimetry assay. In these experiments bacteria were harvested at the late exponential phase. In this assay the anti-NanA serum recognises also to a certain extent glucose grown bacteria (Figure 5A). However in culture media with either ManNAc or NeuNAc as the sole carbon sources, the number of NanA expressing bacterial cells significantly increased reaching 73.7% (± 3.4) and 79.6% (± 4.9), respectively. Differences in NanA production between bacterial cells grown with either of the two amino sugars and control cells cultured in glucose or glucose plus ManNAc were statistically significant (Figure 5A). For more detailed analysis a fluorescence assay for the detection of cell surface-associated neuraminidase activity was carried out to investigate the dependence of neuraminidase production upon the nature of the carbon source provided during bacterial growth (glucose *vs* N-acetylmannosamine). The amount of neuraminidase activity in cell samples containing 10^7^ CFU/ml was clearly higher (approx. 12-fold) in the presence of N-acetylmannosamine rather than glucose (Figure 5B), indicating that N-acetylmannosamine is an inducer of neuraminidase production while in glucose grown cells neuraminidase activity is clearly repressed. 10^7^ CFU of *S. pneumoniae* FP65 grown in the presence of N-acetylmannosamine yielded a neuraminidase activity equivalent to that of 7.5 μg of purified NanA, indicating that this strain produces a significant amount of neuraminidase(s) in the presence of amino sugars. These numbers are such to propose approximately 100–500 enzymes per cell when bacteria are grown in amino sugar and only few enzymes per cell when bacteria are grown in glucose.

## Discussion

Pneumococcal neuraminidases are the most studied surface located glycosyl-hydrolases due to their role in pathogenicity as factors involved in adhesion and invasion of *S. pneumoniae* to host cells [5,6,8,9,27,28]. In addition, their role in the release of free sialic acid from oligosaccharides has been proposed as an important source of carbon and energy [13,14,29,30]. More recently, the cleavage of sialic acid from O-glycans has been related to pathogenesis, by proposing sialic acid as a molecular signal to promote in vitro biofilm production and in vivo nasopharyngeal carriage and lung invasion by *S. pneumoniae*[10,17]. In this context, the regulatory mechanisms of the neuraminidase locus expression are of importance. So far nearly all data on virulence and expression of the two loci containing neuraminidases has been carried out on the *nanAB* locus only, since the D39 reference strain does not carry the *nanC* locus [18]. The main finding on expression of the *nanAB* locus reported its organisation in four predicted transcriptional units, of these the one harbouring NanA and the one encoding for the enzymes of the sialic acid metabolism were differentially expressed in transparent and opaque pneumococcal colony variants [21]. Additionally the increased expression of this locus during infection [10,24,25], further underlines the importance of neuraminidases in the interaction of pneumococci with the host. It should be noted that most of the above work on pneumococcal virulence is done utilising strain D39, which is unable to ferment sialic acid due to a frame shift in the neuraminate lyase of the *nanAB* locus [23,31], a fact which apparently does not influence regulation of the locus and virulence of the bacterium.

We have recently shown that the two ABC transporters of the *nanAB* locus, and also the sodium symporter of the *nanC* locus to a lesser extent, are not only involved in sialic acid uptake, but also in the transport of ManNAc, which represents the first metabolic intermediate in pneumococcal NeuNAc catabolism [23]. In this work we focus our attention on the contribution of the *nanAB* locus, since deletion mutants for the *nanC* locus had been shown not to influence growth on ManNAc and NeuNAc during the first 18–24 hours of incubation, implying a limited or absent regulatory crosstalk between the two regulons [14,23]. The two ABC transporters were shown to be able to support growth on amino sugars, with SPG1596-8 and SPG1589-91 being the main transporters for ManNAc and NeuNAc, respectively [23]. In this work we have combined genomic information, gene expression and growth phenotypes to further clarify these data. When performing *in silico* analysis of the *nanAB* locus we observed the presence of part of the locus in related oral streptococci. Here we utilised this genomic information to strengthen the correlation between orthologous transporters and metabolic functions. *S. sanguinis* and *S. gordonii*, harbouring an operon including the orthologue of the SPG1596-8, were found to be able to efficiently metabolise ManNAc, but not NeuNAc. To the contrary *S. mitis* and *S. oralis*, which are much more closely related to pneumococci, harboured a locus, in addition to all the metabolic genes, also encoding for a neuraminidase and the orthologue of the *satABC* SPG1589-91 transporter [14]. The finding that *S. mitis* can efficiently metabolise NeuNAc and ManNAc, confirm that the substrate specificity identified for the pneumococcal transporters is generally well conserved in orthologues of related species [14]. Interestingly, all oral streptococci share the core part of metabolic enzymes of the operon, suggesting comparable capability to metabolise both NeuNAc and ManNAc. These observations match earlier data that described detectable levels of metabolism of NeuNAc in most oral streptococci, while sialidase activity could only be found in few species [32]. Amongst the oral streptococci, pneumococci carry a composite locus, probably assembled from the gene pool of related species. The association of the SPG1594 oxidoreductase with ManNAc metabolism and of two small hypothetical proteins (SPG1586 and SPG1588) with NeuNAc metabolism remains unexplained, as all necessary enzymes for sialic acid metabolism appear to be already present. The PTS transporter, found to transport glucosamine, appears to be unique in pneumococci [23]. The fact that glucosamine is the last metabolic intermediate in sialic acid catabolism may indicate a convenience for the bacterium in co-utilisation of GlcN and ManNAc, even if it is not clear where pneumococci should feed on GlcN, a rare sugar in the human nasopharynx, but of which on the contrary the pneumococcal cell wall is exceptionally rich [33].

When pneumococci grow on ManNAc and NeuNAc as the sole carbon sources, the generation time is much longer than on glucose or on the yeast-extract derived carbohydrates of the CAT medium, which is in accordance with previous data [23]. Growth on ManNAc (Figure 3B, Figure 4A) shows a profile with a change in generation time. In the case of growth on glucose repression of the whole locus indicates sequential utilisation of sugars. This is less clear for the growth on yeast extract derived dextran and ManNAc, where only part of the locus is induced with the exception of the predicted central transcriptional unit encoding the principal ManNAc ABC transporter SPG1596-8. The data here presented thus do not rule out, that during growth on yeast derived sugars also ManNAc may be co-metabolised. The differential impact of regulation on the three operons is reminiscent of data on expression of this locus in transparent colony variants, where also the *nanB* and ManNAc-uptake operon is not involved in differential expression, while the other two transcripts are upregulated [21]. The fact that both ManNAc and NeuNAc are able to efficiently induce the operon is in accordance with our finding that the SPG1583 regulator acts a positive regulator, as documented by absence of metabolism in its mutant and also by its annotation as a phosphor-sugar binding regulator. Since NeuNAc is imported by an ABC transporter, which does not phosphorylate during uptake, and is first hydrolysed to ManNAc before becoming phosphorylated (Figure 1B), both amino sugars may equally originate the inducer of the positive regulator; probably ManNAc-phosphate.

The sequential utilization of carbon sources is generally regulated by carbon catabolite repression, and in bacteria it has been linked not only to metabolic use, but also to more general mechanisms involved in host-pathogen interactions [34,35]. As in other Gram-positive bacteria, also in *S. pneumoniae* carbon catabolite repression involves the catabolite control protein A (CcpA) which regulates operons by binding to a specific operator sequence, named as catabolite-repressible element (*cre* site) [36-39]. Multiple *cre* sites were recently predicted upstream SPG1601, SPG1597 and SPG1593 in the *nanAB* locus [37,38], and array analyses proved the role of CcpA in its regulation and interestingly relief of *ccpA* repression shows much more pronounced effects on the “NeuNAc-operon” (SPG1593-84) than on the “ManNAc operon” (SPG1599-4). The *cre* sites and CcpA-mediated regulation is in accordance with the transcriptional units described earlier [21]. Our data here confirm that glucose completely represses the expression of all three predicted transcriptional units of the *nanAB* locus. The above gene expression data are also consistent with the neuraminidase activity assay on whole cells, which indicates twelve times more enzymatic activity in induced cells with respect to glucose grown cells. The repression of both neuraminidases and the intracellular enzymes for sialic acid metabolism had already been reported for a large number of viridians streptococci, which thus share with *S. pneumoniae* a strong effect of carbon catabolite repression on the loci responsible of NeuNAc metabolism [32].

## Conclusions

In summary, the data obtained in our study confirmed and demonstrated that, (i) pneumococci carry a composite locus, in part shared by related species, which is predicted to metabolise both ManNAc and NeuNAc, (ii) pneumococci could use both ManNAc and NeuNAc as the sole carbon sources for growth, (iii) uptake of ManNAc and NeuNAc involved preferentially the SPG1596-8 and the *satABC* SPG1589-91ABC transporters, respectively, (iv) ManNAc and NeuNAc could induce the *nanAB* locus, which is subjected to carbon catabolite repression by glucose and (v) a quantitative neuraminidase activity assay allowed to tentatively quantify neuraminidases on the surface of pneumococci grown in amino sugars to numbers around 100–500 enzymes per cell. Interestingly, some growth conditions were found to mimic the transcriptional profile observed for pneumococcal transparent colony variants, suggesting a metabolic influences on pneumococcal phase variation [21]. Still, the differential induction of the predicted transcriptional units by the two amino sugars, indicates that probably carbon catabolite repression and activation by the regulator act at different strength on the three transcriptional units. Finally as already shown in oral streptococci [32], the amount of NanA significantly increases and neuraminidase activity during growth on ManNAc or NeuNAc, indicating that experimental conditions based on mid log glucose-grown bacterial cells may be biased in estimating the actual contribution of neuraminidases to host-pathogen interaction.

## Methods

### Bacterial strains and culture media

Bacterial strains used in this work were *S. pneumoniae* strain G54 (serotype 19F) and its un-encapsulated derivative FP65 [40], since the pneumococcal reference strain D39 has a frame shifted neuraminate lyase gene and TIGR4 did not grow efficiently in CAT medium [23]. Most experiments are performed with the un-encapsulated FP69 as strains without are non virulent and no influence on sugar metabolism has been observed (data not shown). Oral streptococci where *S. mitis* NCTC12261 (kindly provided by Morgens Kilian) and *S. gordonii* V288 Challis [41]. Bacteria were plated on Tryptic soy agar plates (TSB; Liofilchem Roseto degli Abruzzi, Italy) containing 3% v/v of horse blood. Stocks grown in TSB at 37°C to OD_590_ of 0.2 were supplemented with 20% glycerol and stored at −80°C. For fermentation assays and growth curves, bacteria were grown in CAT medium composed of bacto casitone 10 g/l (Becton Dickinson), bacto yeast extract 1 g/l (Becton Dickinson), tryptone 5 g/l (Oxoid Hampshire, UK) and sodium chloride 5 g/l [42]. Just before use, CAT medium was supplemented with 3% w/v of K_2_HPO_4_ 0.5 M [43], a carbon source and catalase 200 U/ml. The sugars were glucose (Sigma-Aldrich), N-Acetylneuraminic acid (NeuNAc, Carbosynth, Compton, UK) and N-Acetyl-D-mannosamine (ManNAc, Carbosynth, Compton, UK). Due to the presence of bacto-yeast extract (Beckton Dickinson), the carbohydrate non-supplemented CAT medium contained 0.16 g/l of total carbohydrate.

### Mutant construction

Mutants were constructed by direct transformation of *S. pneumoniae* with PCR generated recombinant DNA fragments [43]. For deletion of the whole *nanAB* locus, primers NanA1 (TGTAGCCGTCATTTTATTGCTAC), NanA2 (TCCACTAGTTCTAGAGCGATTTTCTGCCTGATGTTGGTAT), NanA3 (ATCGCTCTTGAAGGGAATGCTATTTACACCATACTTCCT), and NanA4 (CAGCTTCGCCTTGCCGTAGGT) were used to amplify segments to allow the integration of the spectinomycin marker *aad9* and the deletion of the whole *nanAB* locus (SPG1583 -SPG1600). For deletion of the SPG1583 regulator, primers DC_09 (TGTCTACGATAGCCGTTGAG), DC_10 (ATCAAACGGATCCCCAGCTTGAACCAGCATCATGGATGAAAATTG), DC_11 (ATATTTTACTGGATGAATTGTTTTAGAAAGCCGTCTTGGTCTGTC), and DC_12 (AATCGCTCGCTATTTTTTGC) were used to amplify segments to allow the substitution of the kanamycin maker *aphIII* with the whole *nanAB* locus, as previously described [44].

### Bioinformatic tools

Comparative genomic analysis was performed using the ACT (Artemis Comparison Tool) [45]. Genbank files for sequence comparison were downloaded directly from the NCBI website. The *S. pneumoniae* genomes utilised were of strain TIGR4 and G54 (NC_003028 and NC_011072). The genomes used for comparison were from *S. gordonii* strain Challis (NC_009785) [46], *S. mitis* strain B6 [47] (NC_013853), *S. oralis* strain Uo5 (NC_015291) [48], and *S. sanguinis* strain SK36 (NC_009009) [49].

### Carbohydrate fermentation

The method for evaluation sugar uptake and fermentation has been recently described [23]. Briefly, bacteria grown on agar plates were resuspended to an OD_590_ = 0.6 in CAT medium and diluted 1:1 with CAT medium supplemented with K_2_HPO_4_,the appropriate sugar and catalase as reported above. After o.n. incubation, pH changes were visualised by addition of phenol red (0,1 mg/ml) (P4633 Sigma-Aldrich).

### Growth curve and sample collection

In order to characterize the gene expression pattern in a specific point of the growth curve, we sampled bacteria during growth. Strains were grown on TSA plates at 37°C in a CO_2_ enriched atmosphere for 18 hours. Bacteria were then collected with a swab and resuspended at the OD_590_ of 0.2 in non-supplemented CAT medium. Bacterial samples were diluted 1:100 in CAT medium either without added sugar or with addition of either glucose, ManNAc, NeuNAc, glucose + ManNAc, or glucose + NeuNAc, all at 1 g/L. Bacterial growth curves were performed in 96-well plates in a thermostated spectrophotometer at 37°C. Plates were shaken gently for 10 seconds prior to each reading, and the optical density was read automatically in 10 min intervals at a wave length of 590 nm. Triplicate samples were collected from microwells for gene expression analysis and cytofluorimetry. For RNA extraction and retrotranscription, the samples were transferred to microtubus, centrifuged at 13000 rpm at 4°C for 1 min, and the pellet was conserved at −20°C. For flow-cytometry analysis, the samples were centrifuged at 8000 rpm at room temperature for 5 min and immediately analysed.

### RNA extraction, retrotranscription and qPCR

RNA was extracted using the NucleoSpin RNA II kit (Macherey-Nagel) according to the manufacturer’s instructions, and the RNA samples were frozen in aliquots until use. cDNA synthesis was carried out using the Transcriptor First strand cDNA synthesis kit (Roche) according to the manufacturer’s instructions. Annealing was performed at 25°C for 10 min, extension at 37°C for 1 h, and finally inactivation at 70°C for 15 min. The qPCR was performed as previously described [50], by mixing 2 μl of cDNA template, 10 pmol of primers, and 2 μl of Light Cycler DNA-Master SYBR Green I (Roche). The reaction was carried out in a Light Cycler apparatus (Roche). Primer efficiency was verified by serial dilution of cDNA ranging from 10^2^ to 10^6^ target copies per reaction. Primers were designed on *gyrB* (reference gene; CAGATCAAGAAATCAAACTCCAA and CAGCATCATCTACAGAAACTC), *nanA* SPG1600 (AGCAACCTCTGGCAAATGAA and ATAGTAATCTCTTGGAATT), SPG1598 (GGTCAACTCAGATGCTT and GAGGAACAGAGTAGTAATC), SPG1592 (CCAACCACGATAGCAAC and CTGAATACAACCTCTCC) and SPG1591 (CAGGTGCTTTCCCAGTC and GTGTTGTAGTATGGTGAG) [24,50]. The relative gene expression was analysed by using the 2^–ΔΔCT^ method [51]. At least three replicas were used for any given sample. Statistical analysis was conducted by using the two-tailed Student *t* test.

### Flow cytometry assay

FP65 pneumococci grown in media with carbohydrate supplementations at 1 g/L to late log phase were resuspended in 500 μl of phosphate-buffered saline (PBS; pH 7.4) containing 1% bovine serum albumin and incubated at 37°C for 30 min. Samples were spun down and pellets were resuspended in anti-NanA rabbit serum diluted 1:100 in PBS/BSA and incubated at 4°C for 1 h (negative controls were incubated without antibody). After two washes with 1 ml of PBS, 100 μl of fluorescein isothiocyanate (FITC)-conjugated anti-rabbit (1:64; Sigma-Aldrich) was added to bacterial pellets. The resuspensions were incubated at 37°C for 30 min and then washed twice in PBS. Samples were finally resuspended in 300 μl of paraformaldehyde 1% in PBS and subjected to flow cytometry (FACScan, Becton Dickinson, San Diego, CA). Statistical analysis was carried out by using two-tailed Student *t* test.

### Neuraminidase activity

The neuraminidase activity was measured using the fluorogenic substrate 2′-(4-methylumbelliferyl)-α-D-N-acetylneuraminic acid (4MU-Neu5Ac) (M8639, Sigma-Aldrich, St. Louis, Miss.). The time dependence of the variation of fluorescence (λ_excitation_, 335 nm; λ_emission_, 400 nm) in the presence of cell or enzyme samples was recorded with a EnVision multilabel plate reader (Perkin Elmer, Waltham, Mass.) using 50 μM 4MU-Neu5Ac in 10 mM MES buffer at pH 6.0, in a final reaction volume of 200 μl. *S. pneumoniae* FP65 was grown in CAT medium, containing alternatively glucose or N-acetylmannosamine as the carbon source, respectively, for 18 hours at 37°C. The sample was prepared as follows; the culture was centrifuged at 10,000 × g (4°C) and the cell pellet washed once in an equal volume of 10 MES buffer pH 6.0, centrifuged and resuspended at a final A^600^ = 0.4 in 10 mM MES pH 6.0. The method was initially optimized and calibrated using purified NanA neuraminidase of *S. pneumoniae* D39 produced in *E. coli* (0.88 mg/ml) (data not shown). The activity was computed as the variation of fluorescence *vs* time using a linear regression of the data. In our conditions, 1 μg of purified NanA yielded a activity of 10,690 ΔF/min.

## Abbreviations

NeuNAc: Sialic acid (N-acetylneuraminic acid); ManNAc: N-acetyl mannosamine; ABC transporter: ATP binding cassette transporter; PTS transporter: Phosphotransfer system transporter; CAT medium: Casitone yeast extract medium.

## Competing interests

The author declare that they have no competing interests.

## Authors’ contributions

LG, JKH, AG, AB, LC, and CT generated data in the laboratory and implemented the project under the supervision of GP, JDD, PWA, SR and MRO. All authors contributed to the writing of the final manuscript. All authors read and approved the final manuscript.
